# Research progress on the role of hormones in ischemic stroke

**DOI:** 10.3389/fimmu.2022.1062977

**Published:** 2022-12-07

**Authors:** Shuyuan Huang, Lu Liu, Xiaodong Tang, Shulan Xie, Xinrui Li, Xianhui Kang, Shengmei Zhu

**Affiliations:** ^1^ Department of Anesthesiology, the First Affiliated Hospital, College of Medicine, Zhejiang University, Hangzhou, Zhejiang, China; ^2^ Department of Anesthesiology, Shenzhen People’s Hospital, The Second Clinical Medical College, Jinan University, The First Affiliated Hospital, Southern University of Science and Technology, Shenzhen, Guangdong, China

**Keywords:** ischemic stroke, hormones, immunomodulation, brain protection, mechanisms

## Abstract

Ischemic stroke is a major cause of death and disability around the world. However, ischemic stroke treatment is currently limited, with a narrow therapeutic window and unsatisfactory post-treatment outcomes. Therefore, it is critical to investigate the pathophysiological mechanisms following ischemic stroke brain injury. Changes in the immunometabolism and endocrine system after ischemic stroke are important in understanding the pathophysiological mechanisms of cerebral ischemic injury. Hormones are biologically active substances produced by endocrine glands or endocrine cells that play an important role in the organism’s growth, development, metabolism, reproduction, and aging. Hormone research in ischemic stroke has made very promising progress. Hormone levels fluctuate during an ischemic stroke. Hormones regulate neuronal plasticity, promote neurotrophic factor formation, reduce cell death, apoptosis, inflammation, excitotoxicity, oxidative and nitrative stress, and brain edema in ischemic stroke. In recent years, many studies have been done on the role of thyroid hormone, growth hormone, testosterone, prolactin, oxytocin, glucocorticoid, parathyroid hormone, and dopamine in ischemic stroke, but comprehensive reviews are scarce. This review focuses on the role of hormones in the pathophysiology of ischemic stroke and discusses the mechanisms involved, intending to provide a reference value for ischemic stroke treatment and prevention.

## Introduction

Ischemic stroke is a neurological disorder caused by the disturbance of blood supply to the brain (1). Globally, stroke was the third leading cause of death after neonatal diseases and ischemic heart disease in 2019, accounting for more than half of new strokes (2). Low-income countries bear a greater disease burden than high-income countries (2). From 1990 to 2019, the incidence of ischemic stroke has increased significantly in China (3). Treatment options include tissue plasminogen activator (tPA) and mechanical thrombectomy (MT) (4). However, these are limited by the narrow treatment-time window (4). Endovascular thrombectomy and intravenous thrombolysis (IVT) combined with drug therapy have been popular treatment regimes in recent years (5–7). However, less than 5% of acute ischemic stroke patients receive IVT within the eligible treatment window, and fewer than 100,000 MTs were performed worldwide in 2016 (8). Nevertheless, complications such as cerebral hemorrhage, vessel re-occlusion, and cerebral edema arise after MT (9). Therefore, further research is required on the prevention and treatment of stroke.

Hypothalamus serves as an endocrine organ. It secretes regulatory factors, acts on the pituitary anterior lobe cell, and stimulates the secretion of hormones that control the endocrine glands (10). Hormones transmit information to intracellular by binding to specific receptors inside the cell or on the plasma membrane (11). In the 1970s, there were some reports about the role of dexamethasone in ischemic stroke (12, 13). However, these reports were primarily negative, probably because the concentration of dexamethasone at the site of action was too small to achieve a therapeutic effect (14). Success can only be expected if a sufficiently high dose of dexamethasone is administered immediately after an ischemic attack (14). Since the 1990s, the study of hormones in an ischemic stroke has become popular. Insulin (15), estrogen (16), progesterone (17), testosterone (18), arginine vasopressin (19), and thyroid hormone (20) have been reported successively in ischemic stroke, which gradually fills the gap of hormones in the field of ischemic stroke research. Specific hormonal changes are a risk factor for ischemic stroke (21). Moreover, ischemic stroke can cause hormonal changes (22). Brain damage after stroke results from a complex series of pathophysiological events like excitotoxicity, oxidative and nitrative stress, inflammation, and apoptosis (23). Our study describes the mechanism of the hormones involved in the pathophysiological process of ischemic stroke and gives ideas on the prevention and treatment of ischemic brain injury.

## Thyroid hormone and ischemic stroke

Meta-analysis studies associate the thyroid hormone with the prognosis of ischemic stroke (24). Patients with low initial triiodothyronine (T3) are linked with worse acute ischemic stroke outcomes (25). At the same time, serum thyroid stimulating hormone levels are negatively correlation to the risk of post-stroke patient fatigue in the acute phase and follow-up assessment (26). Thyroid hormone improves neurological outcomes after experimental stroke through different pathways, such as being anti-edema (27), promoting the expression of neurotrophic factors (28), regulating neuronal plasticity (29), and increasing adenosine triphosphate (ATP) production (30). Simultaneously, reversing T3 (rT3) increases neuronal survival after ischemia-reperfusion injury in rat models since it reduces brain metabolism (31). Published review has demonstrated that thyroid hormone-regulated genes are associated with neuronal plasticity after ischemic stroke (32). Recent data suggest that astrocytes are sensitive to T3, and their response to T3 is related to their maturity, for a total of 117 genes are regulated by T3 transcription (33, 34). Astrocytes play a significant role in thyroid hormone deiodination (35), a process affected by ischemic stroke (36). Type 2 iodothyronine deiodinase (D2) is the primary source of plasma T3 in normal thyroid function (37). In the astrocytes, D2 deiodinases T4 to form T3, exerting thyroid hormone effects on other nerve cells in the brain (38). The D2 mRNA expression was upregulated in the ipsilateral striatum after 6h of rat middle cerebral artery occlusion and disappeared after 24h (36). In the ipsilateral cortex, the D2 mRNA was induced at 6h; increased at 24h and decreased at 72h (36). A similar situation was found in the rat traumatic brain injury, where the astrocytes’ D2 mRNA expression was upregulated (39).

Retrospective studies show that Low T3 predicts poor functional prognosis in patients with acute ischemic stroke and is more significant in the elderly (40, 41). Further, T3 infusion promoted D2 gene expression in risk areas in cardiac ischemia-reperfusion models (42). After T3 infusion, serum T3 levels in the tested risk area are the same as the basal level (42). We hypothesize that ischemic stroke promotes D2 expression in astrocytes, thereby promoting the deiodination of T4 to T3. T3 then promotes D2 expression, forming a positive feedback loop. This cascade contributed to the recovery of T3 levels, and the protective effect of T3 was exerted in ischemic stroke.

## Thyroid hormone derivatives and ischemic stroke

3-Iodothyroamine (T1AM) is a derivative of endogenous thyroxine (43). T1AM is derived from the enzymatic digestion and decarboxylation of T4 (43). Studies indicate that T1AM biosynthesis depends on the sodium-iodine transporter and thyroid peroxidase (44). In the mice’s intestinal tissues, T4 forms T1AM by decarboxylation of ornithine decarboxylase and subsequent deiodination (45). In a mouse model, T1AM reduced the infarct size by inducing hypothermia (46). Meanwhile, T1AM was used as an antecedent treatment to induce neuroprotection from subsequent ischemia (46). Hypothermia is believed to be due to peripheral vascular dilation and subsequent heat loss (47). T1AM induced tail vessel dilation in male mice through the hypothalamus signaling pathway (47).

Hypothermia is a feasible treatment for stroke (48). Preclinical studies have recognized the protective role of hypothermia in ischemic stroke (49). Moderate hypothermia reduces the inflammatory response Interleukin 1 beta (IL-1β) and Tumor Necrosis Factors alpha (TNF-α), oxidative stress (50), and energy consumption (51) after an ischemic stroke. Recent progress has been observed in studies involving low temperature combined with other neuroprotective measures (anesthetics, psychotropic agents, antibiotics, oxidative stress scavengers) (52). Reducing the surface temperature to 35°C was possible in conscious patients with acute ischemic stroke, but cooling was associated with the risk of pneumonia (53). Combining intra-arterial recanalization with isotonic saline infusion (4°C) in the ischemic area using an angiographic catheter reduced the ischemic area temperature by at least 2°C; the body temperature decreased slightly (up to 0.3°C) (54). No intracerebral complications associated with hypothermia were observed (54). Intravascular hypothermia circumvented the core hypothermia and reduced the risk of pneumonia associated with systemic hypothermia. Preclinical studies indicate the protection offered by T1AM on ischemic stroke by inducing hypothermia. However, further studies are required to determine the clinical utility.

## Growth hormone and ischemic stroke

Additional clinical investigations are required to conclude the effects of growth hormone (GH) on ischemic stroke. Patients with a stroke are at risk for growth hormone deficiency (55). Agonistic analogs of growth hormone-releasing hormone are beneficial in mouse ischemic stroke (56). Low GH is common after severe ischemic stroke patients, and GH may be related to the prognosis of ischemic stroke (57).

### Growth hormone improves motor function after ischemic stroke

Growth hormone (GH) has a nutritional effect on the nerves (58). It functioned as an effective neurotrophic factor for the inner ear neurons and significantly increased neurite extension and neuronal branching of rat spiral ganglion cells (59). It also repaired nerves (60). In the chronic denervation injury model, GH showed robust nerve regeneration through axon density, axon diameter, and myelin sheath thickness (61). At the same time, GH improved muscle innervation and reduced muscle atrophy (61). Randomized controlled trials demonstrated that human growth hormone improves quadriceps atrophy and deficiency drop after Anterior Cruciate Ligament (ACL) reconstruction and increases quadriceps strength in patients (62). GH improved motor function after an experimental stroke, as demonstrated by the cylinder and grid walk tests (63). This is associated with GH promoting increased cell proliferation, neurogenesis, synaptic plasticity, and angiogenesis within the peri-infarct region (63). GH also increased insulin growth factor 1 (IGF-1). After GH treatment, a significant positive correlation existed between plasma IGF-1 levels and cylinder task performance (63). In an ischemic stroke rat model, IGF-1 plays multiple roles in increasing sensorimotor function, improving cognitive function, and reducing infarct size (64–66). Patients with higher serum IGF-1 were significantly associated with a lower risk of ischemic stroke (67). Compared with the same shuttle vector, female rats carrying the IGF-1 gene exhibited better sensorimotor function in the early and late acute stages of stroke (68). In conclusion, GH improves motor function after stroke through its neuromuscular nutrition and repair function. Additionally, it improves motor function by increasing IGF-1.

### Growth hormone improves cognitive function after ischemic stroke

The prevalence of cognitive impairment in stroke survivors ranges from 20% to 80%, depending on country, ethnicity, and diagnostic basis (69). Stroke was associated with a sharp decline in cognitive performance that accelerated and continued over the next few years (70). At the same time, patients with cognitive impairment have a higher risk of future stroke than those with normal cognitive function (71). Post-stroke cognitive impairment as an independent predictor of ischemic stroke recurrence (72). Hippocampal atrophy was related to cognitive impairment in Alzheimer’s disease (73), Lewy’s dementia (74), small vascular disease (75), type 2 diabetes (76), and Parkinson’s dementia (77). The hippocampal atrophy rate was higher in the stroke participants than in the control group, and the hippocampal atrophy rate was higher in the early stage than in the late stage (78). Also, more severe atrophy was observed in the CA1 region of the hippocampus and caudal hippocampus in ischemic stroke patients (79). However, a study demonstrated that long-term cognitive impairment in ischemic stroke patients was associated with hippocampal deformation, not atrophy (80). Resting-state functional magnetic resonance imaging has shown that reduced hippocampal-subparietal lobule connectivity is associated with cognitive impairment in patients with ischemic stroke (81). In summary, cognitive impairment after ischemic stroke was closely related to the hippocampus.

GH therapy may play a role in improving cognitive function (82). In patients with an isolated growth hormone deficiency, white matter abnormalities in the corpus callosum and corticospinal tracts and reduced thalamic and globus pallidus volumes are associated with deficits in cognitive function and motor function performance (83). In older rats, age-related reductions in growth hormone lead to cognitive decline, partly through changes in short-term hippocampal plasticity (84). GH treatment enhanced the regulation of excitatory synaptic transmission and plasticity in the aged rat hippocampus by activating N-methyl-D-aspartate receptor (NMDAR)-dependent basal synaptic transmission and alpha-amino-3-hydroxy-5-methyl-4-isoxazolepropionate receptor (AMPA-R)-dependent basal synaptic transmission, which altered the course of cognitive decline (85). GH increases the density of dendritic spines in the hippocampus, thus strongly influencing hippocampal plasticity and memory (86, 87). Randomized controlled trials demonstrated the beneficial effects of recombinant human growth hormone on cognitive impairment after stroke (88). Mice treated with GH after a stroke had a more remarkable ability to complete paired associative learning tasks (89). This ability was associated with GH increasing the neurotrophic factors (IGF-1, Vascular endothelial growth factor (VEGF)) and promoting synapses, myelin, and brain vascular network formation (89). GH also increased hippocampal-dependent visual discrimination in male mice after experimental cortical stroke, which was associated with GH stimulation of neural progenitor cell proliferation, increased synaptic plasticity in the hippocampus, and increased plasma IGF-1 levels (90). Thus, GH improved cognitive function after ischemic stroke *via* the hippocampus.

## Sex hormones and ischemic stroke

### Testosterone and ischemic stroke

Serum testosterone was reduced after acute ischemic stroke in men, and total testosterone negatively correlated with infarct size (18). Low testosterone levels were associated with an increased risk of ischemic stroke in older men (91, 92) and possibly higher all-cause mortality after acute ischemic stroke (93). Also, anger tendencies and emotional incontinence after ischemic stroke were related to low testosterone levels (94). However, in the pediatric population, increased testosterone elevates the risk of stroke (95). The effect of testosterone on ischemic stroke was age-dependent. Testosterone exacerbated ischemic brain injury in young adult mice, while testosterone supplementation reduced cortical infarction in middle-aged mice (96). This protection was mediated by androgen receptors (AR) and unrelated to the brain aromatase (96). AR expression was reduced after cerebral ischemia, and overexpression of AR reduced the infarct size after ischemic stroke (97). Interestingly, exposure to testosterone during neonatal life in adult male rats increased their resistance to ischemic stroke (98). The upregulated testicular aromatase expression increased the serum estradiol levels, which exerts a protective effect by increasing X-linked apoptosis inhibitors (98). Also, supplementation of testosterone in middle age rats to the normal physiological levels of young male rats reduced infarcts (96).

However, testosterone can be detrimental to ischemic stroke. Dihydrotestosterone (DHT) suppresses peripheral immunity after ischemic stroke (99). DHT eliminates the presence of immature neurons in the ischemic region and reduces the repair of damaged tissue after ischemia (100). More research is required for applying testosterone replacement therapy (TRT) to ischemic stroke (101). In older men with low testosterone levels, TRT increases the risk of cardiovascular events, especially in the first two years of use (102). However, TRT reduced the risk of cardiovascular outcomes in androgen-deficient men during a median follow-up of 3.4 years (103). Further research on testosterone is warranted, including its therapeutic effects on different age groups, the mechanism of its protection, and its role as a prognostic predictor of ischemic stroke.

### Estrogen and progestin with ischemic stroke

The Women’s Health Initiative (WHI) showed that estrogen (E) plus progestin (P) increased the risk of ischemic stroke in generally healthy post-menopausal women (104). However, altering the route of hormone administration and the type of hormone may remedy this drawback. Encouraging hormone therapy users to switch from oral to transdermal estrogen and from synthetic to micronized progesterone reduced the risk of ischemic stroke by ≤ 3000 per million hormone therapy users per year (105). Meanwhile, using E and P in combination has progressed in the preclinical study of ischemic stroke. Combined E and P treatment reduced cortical infarct size in rats suffering from ischemic stroke (106–108). Combined E and P treatment inhibited ischemia-induced neuronal apoptosis by suppressing Calpain-1 upregulation and caspase-3 activation in rat cortical infarct areas (109). E plus P also reduced the extracellular glutamate levels by inducing the glutamate transporter protein (glutamate transporter 1 (GLT-1) and amino-acid transporters (EAAT3)) expression in an ischemic stroke rat (110). The neuroprotective role of E and P in stroke may be due to reduced phosphorylation of the heat shock protein 27 (HSP27) in rat ischemic areas (111). 17β-estradiol plus P displayed anti-inflammatory effects by selectively reducing absent in melanoma 2 (AIM2) and NLR family CARD domain-containing protein 4 (NLRC4) inflammasomes in primary cortical astrocytes and microglia after ischemic stroke in rats (112).

After transient middle cerebral artery occlusion in rats, E plus P regulated chemokine-microglia/lymphocyte interactions, a mechanism associated with cytoprotection (113). E plus P attenuated the expression of ischemic stroke-induced proinflammatory chemokines chemokine ligand 2 (CCL2), chemokine ligand 5 (CCL5), and interleukin 6 (IL-6) (113). Moreover, the local expression of microglia/macrophage/lymphocyte markers (ionized calcium -binding adapter molecule 1(Iba-1), cluster of differentiation 8 (CD8), and cluster of differentiation 3 (CD3)) in the penumbra areas was significantly reduced after hormone treatment (113). In a rat model, E plus P indirectly regulated pro-apoptotic and inflammatory gene translation by selectively inhibiting miR-223 and miR-214 and further enhancing miR-375 (114).

Further, few studies report the relation between the estradiol/testosterone ratio and ischemic stroke, and they are less optimistic. Increased estradiol and decreased testosterone levels were associated with acute ischemic stroke in male patients (115). For post-menopausal women with a body mass index < 25 kg/m^2^, a higher estradiol/testosterone ratio was associated with a significantly higher risk of ischemic stroke among the patients currently treated with exogenous hormones (116).

### Oxytocin and ischemic stroke

**Figure 1:**
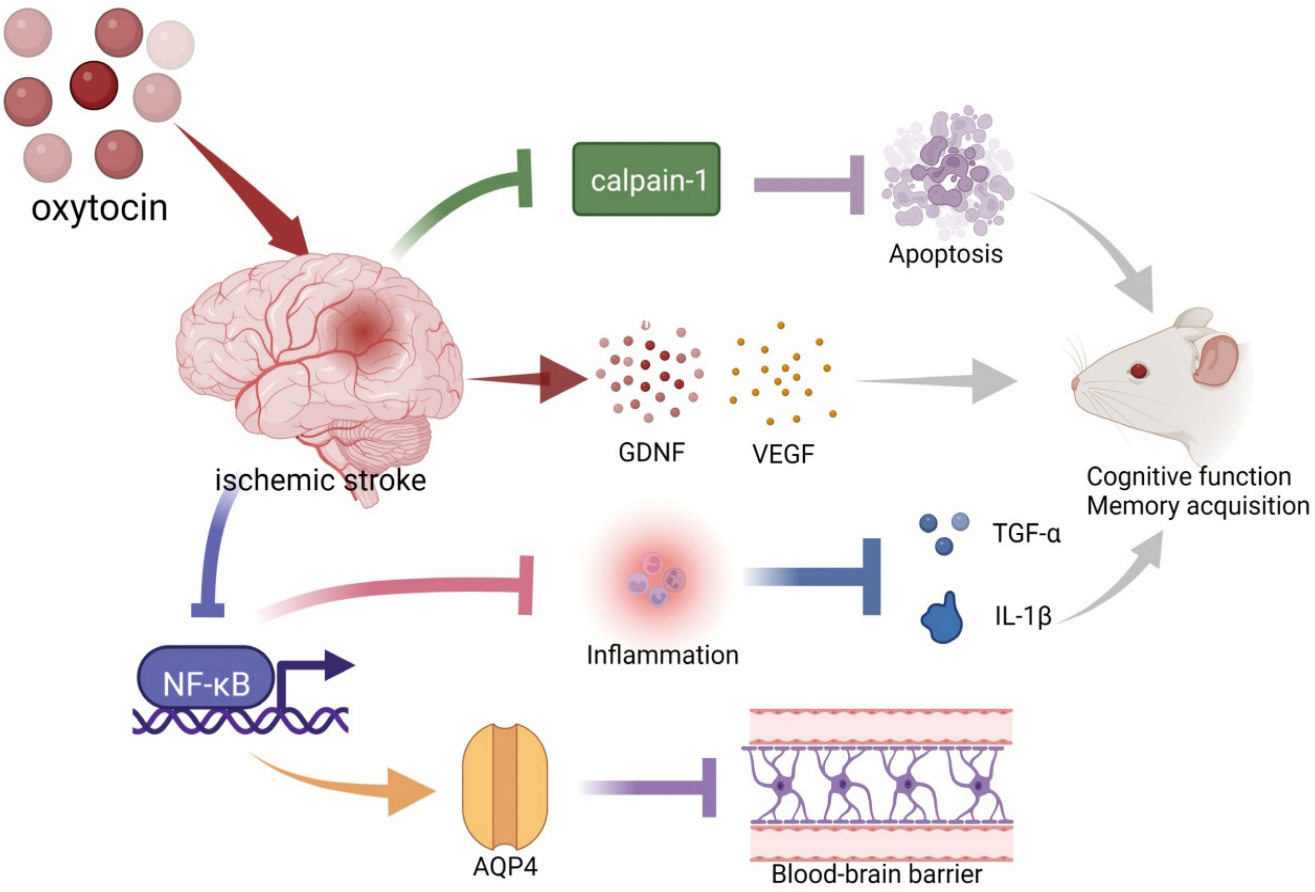
Schematic illustration of oxytocin and ischemic stroke. OT decreased ischemia-induced Caipain-1 overexpression to inhibit apoptosis. Pretreatment with OT before ischemic stroke promoted the expression of BDNF and VEGF. OT suppressed inflammation (TNF-α and IL-1β) by inhibiting the expression of NF-κB. These are beneficial for the recovery of cognitive function after an ischemic stroke. In addition, OT attenuated blood-brain barrier leakage and cerebral hematoma by promoting the expression of AQP4, which may be associated with the inhibition of NF-κB. BDNF, brain-derived neurotrophic factor; VEGF, vascular endothelial growth factor; TNF-α, tumor necrosis factor-α; IL-1β, Interleukin-1 beta; AQP4, Aquaporin 4. The illustration was supported by BioRender (https://biorender.com).

Clinical studies of oxytocin (OT) use in ischemic stroke are scarce, but experimental studies have robust progression (**Figure 1**). OT reduces brain damage after experimental stroke (117–119). Compared with the ischemia control group, OT significantly reduced the infarct volume in the cerebral cortex and striatum (117), thus, improving the spatial memory function (118). Meanwhile, OT pretreatment significantly reduced the number of hippocampal neuronal deaths after focal cerebral ischemia (119). The protective effect of OT on brain injury after ischemic stroke was correlated with the increased expression of VEGF, Aquaporin 4 (AQP4), and Brain-derived neurotrophic factor (BDNF) proteins, reduced leakage from the blood-brain barrier (BBB), decreased inflammatory mediators TNF-α and IL-1β, and reduced cell death and apoptosis (117, 118). In addition, OT ameliorated ischemic stroke by attenuating Calpain-1 (117). Calpain-1 and caspase-3 were positively correlated in ischemic stroke, suggesting that down-regulating calpain-1 inhibited apoptosis (109). Calpain-1-specific inhibitor PD151746 promoted phosphorylated signal transducer and activator of transcription 3 (p-STAT3) expression and was auxiliary to the proliferation and functional recovery of neural precursor cells in the subventricular zone after stroke (120).

### Prolactin and ischemic stroke

Studies on prolactin (PRL) and ischemic stroke are scarce, but reports on brain injury (121) and neuroprotection (122, 123) have seen some advances. PRL mainly exerts neuroprotective effects by inhibiting excitatory toxicity (124, 125) and neuroinflammation (126, 127). In the cerebral ischemia model, PRL reduced the cerebral infarction area and cerebral water content and restored the physiological status (128). Transient ischemic attack increased PRL concentrations and increased plasma PRL levels were significantly linked with platelet P-selectin (129, 130). Platelet surface P-selectin expression was associated with a worsening clinical course in acute ischemic stroke (131). These results suggested that patients with high prolactin levels after ischemic stroke may have a worse prognosis. More research is needed to investigate the prolactin role in ischemic stroke.

## Glucocorticoid and ischemic stroke

Many patients have increased cortisol after acute ischemic stroke, which negatively impacts organ function (132). Ischemic injury to neurons in the rat brain was enhanced by exposure to high physiological titers of glucocorticoid (GC) (133). Pre-hospital GC use increased the 30-day mortality in patients with ischemic stroke (134). Also, the current use of GC increased the risk of myocardial infarction and venous thromboembolism in the first year of ischemic stroke (135). However, a clinical study also indicated an improved level of consciousness in patients with acute ischemic stroke associated with cerebral edema after giving dexamethasone (136). GC resistance was associated with poorer functional outcomes after an ischemic stroke (137).

### GC and ischemic stroke

Many studies suggest that GC is involved in immune regulation in ischemic stroke (138, 139). Intranasal dexamethasone reduced mortality, neurological deficits, infarct size, blood-brain barrier permeability, inflammatory cell infiltration, and glial activation in mice after ischemic stroke (140). In experimental focal cerebral ischemia, dexamethasone was neuroprotective by inhibiting the inflammation-dependent NF-kB-p65 pathway, including the inhibition of inducible nitric oxide synthase (iNOS), Cyclooxygenase-2 (COX-2), TNF-α, and IL-1β expression (141). At the same time, inhibiting the expression of glucocorticoid receptors (GR) significantly increased the expression of proinflammatory cytokines (IL-6, IL-1β, and TNF-α) and decreased the brain-derived neurotrophic factor/pro-myosin receptor kinase B (BDNF/TrkB) signaling in the mice brain, which can increase the infarct size and worsen neurobehavioral deficits in ischemic stroke (142). However, elevated cortisol levels were negatively correlated with blood lymphocyte counts in 20 patients with acute stroke (143). In mice, stroke-induced glucocorticoid release significantly triggered defective B-lymphocyte production (143). Blocking GR prevented post-ischemic lymphocyte reduction (144). Plasma corticosterone levels were elevated in diabetic mice after ischemic stroke (145). Using glucocorticoid synthesis inhibitors reduced the infarct size and IL-6 expression (145). Glucocorticoids are anti-inflammatory and immunosuppressive. Hence, treating ischemic stroke with glucocorticoids is contradictory and complex. More research is needed to maximize the protection of glucocorticoids in ischemic stroke.

## Parathyroid hormone and ischemic stroke

Parathyroid hormone (PTH) and 25-dihydroxyvitamin D levels together can make important contributions to determination of stroke risk (21). PTH levels were elevated in patients with acute ischemic cerebrovascular events (22). In peritoneal dialysis patients, lower serum PTH levels were significantly associated with an increased risk of stroke (146). PTH was beneficial in ischemic stroke. PTH promoted the expression of neuroangiogenesis factors and increased angiogenesis around the infarction after focal cerebral ischemia (147).

Additionally, PTH promoted the migration of bone marrow stem cells (148). Bone marrow-derived endothelial progenitor cells and endothelial stem cells increased in the peripheral blood of stroke mice after PTH treatment (147). These cells highly expressed the migratory chemokine stromal cell derived-factor 1 (SDF-1), which promoted the migration of neuroblasts from the subventricular region to the ischemic cortical region and increased the number of cortical neurons around infarction (147). Meanwhile, parathyroid hormone-related protein (PTHrp) reduced the cortical infarct area in ischemic stroke animals by vasodilating and increasing cerebral blood flow (149). More research is required on parathyroid hormone and ischemic stroke.

## Catecholamines and ischemic stroke

Catecholamines have been linked to an increased risk of infection after stroke (150). Catecholamines increase levels of the pro-inflammatory cytokines IL-1β and Interferon-γ (INF-γ) and decrease levels of the anti-inflammatory cytokine Interleukin 10 (IL-10) after experimental stroke, an immunosuppressive state that lowers the threshold for infection and increases the risk of infection (151). Dopamine release occurs in the early stage of ischemia, and the amplitude of dopamine release correlates with the duration of ischemic injury (152). Reperfusion induces more striatal dopamine release (152). Levodopa is a dopamine precursor, and studies have shown that levodopa is expected to enhance motor recovery after stroke (153–159). Levodopa also enhanced post-stroke plasticity (160). The combination of dopamine precursors significantly reduced the infarct size, proinflammatory cytokine levels, oxidative stress levels, and neurological deficits in the striatum of rats with cerebral ischemia-reperfusion injury (161). Meanwhile, amantadine, a drug promoting dopamine release, improved cognitive and functional recovery after a stroke (162).

### β-adrenergic receptors and ischemic stroke

Pharmacological inhibition of β-adrenergic receptors, but not steroid inhibition, effectively reduced infection and improved clinical outcomes in experimental stroke (163). In a retrospective series of studies, β-blocker use was associated with reduced risk of early death in patients with ischemic stroke (164). β-blocker was negatively associated with the incidence of nosocomial pneumonia before and during the stroke (165). β1 adrenergic receptor of neutrophils is associated with migration during increased inflammation, and β1 adrenergic receptor blocking improves brain damage by targeting neutrophils (166). The β-blocker carvedilol may protect the ischemic brain in the rat by inhibiting apoptosis and attenuating the expression of TNF-α and IL-1β (167). Interestingly, in stroke models, Augmented β2-adrenergic signaling has also been reported as neuroprotective. Unlike systemic administration, central administration of norepinephrine lowers blood pressure and exerting anti-inflammatory and neuroprotective effects (168). Increased β2-adrenergic signaling after an experimental stroke typically inhibits microglial/monocyte-derived macrophage response and reduces the upregulation of pro-inflammatory and anti-inflammatory cytokines (TNFα and IL-10) (169). In mice, increased β2-adrenergic signaling after stroke inhibited post-stroke pneumonia but increased post-stroke infarct size (170).

### Dopamine receptors and ischemic stroke

Cerebral ischemia affects dopamine receptors in the striatum (171, 172) and hippocampus (173). Ischemic dopamine release in the striatum was associated with early transient changes in dopamine receptor-mediated dopamine neurotransmission (172). Cerebral ischemia reduced the number of dopamine D1 receptors (D1R) (171) and also their affinity for receptor ligands (172). Cerebral ischemia slightly affects D2 receptors (D2R) in the striatum for up to seven days (171). Subsequent studies have shown that D2R continued to bind ligands in the first week after cerebral ischemia, declining sharply from day 14 to day 28 (174). These results suggested the critical role of D1R and D2R in the recovery from ischemic stroke.

### D1R and ischemic stroke

D1R activation inhibits the excitatory postsynaptic currents in post-ischemic striatal neurons because it activates Cyclic Adenosine Monophosphate (cAMP)-dependent protein A and adenosine A1 receptors (175). Systemic D1R agonists significantly reduced ischemia-induced striatum cell death after ischemia (175). D1R in astrocytes was also associated with GNDF expression. In the transient middle cerebral artery occlusion (tMCAO) model, adding selective D1R agonists increased GNDF expression, while D1R inhibitors significantly reduced GNDF expression (176). After 2h of ischemia stroke in rats, endogenous tissue fibrinogen activator (tPA) increased in the region of BBB injury, and intrastriatal D1R antagonists significantly reduced ischemia-induced endogenous tPA upregulation and BBB injury (177). Experimental stroke in the dorsolateral striatum induced alcohol preference, enhancing glutamatergic energy input to D1-neurons in the dorsomedial striatum (178). Inhibition of D1R mitigated the stroke-induced increment in the self-intake of alcohol (178).

### D2R/D3R and ischemic stroke

Resident microglia do not express D2R in healthy brains, but this population expresses D2R after cerebral ischemia (179). Dopamine acts as a regulator of microglial function during neuroinflammation, and the D2R/D3R agonist pramipexole enhances nitrite secretion in response to proinflammatory stimuli (179). The D2R agonist bromocriptine prevented ischemia-induced neuron damage in the gerbil by preserving superoxide dismutase (SOD) (180). In the middle cerebral artery occlusion (MCAO) mouse model, Sino suppresses neuroinflammation after ischemic stroke by upregulating D2R/αB-crystallin (CRYAB) expression (181). Also, agonistic D2R induces neurological recovery in ischemia/reperfusion injury following rats *via* the mitochondrial pathway (182). Pramipexole inhibited the transfer of cytochrome C from mitochondria to cytosol, thereby inhibiting the mitochondrial permeability transition pore (182). In the tMCAO rat model, Sumanirole repaired mitochondrial dysfunction by reducing mitochondrial reactive oxygen species production, increasing mitochondrial membrane potential and the activity of protective mitochondrial complexes and histological changes, thereby alleviating ischemic injury (183). Meanwhile, Sumanirole reduced the infarct size, restored behavioral changes, and promoted neuronal survival (183). D2/D3 receptor activation was associated with ischemic preconditioning (IPC), and IPC was beneficial against ischemic reperfusion injury in mice (184). However, compared with D1R on astrocytes, agonistic D2R on astrocytes did not affect the GNDF levels (176).

## Conclusion and future direction

Abnormal hormone levels are typical after an ischemic stroke. Growth hormone and testosterone levels decrease while prolactin, corticosterone, parathyroid hormone, and dopamine levels increase. Also, hormone changes have an effect on the prognosis of ischemic stroke (**Table 1**). Hormones are involved in various pathophysiological mechanisms of ischemic stroke, including cerebral edema formation, neuroplasticity regulation, neurotrophic factor formation, cell death reduction, apoptosis, inflammation, and oxidative stress (**Tables 2** and **3**). It is essential to understand the role of hormones in the pathophysiology of brain injury in ischemic stroke for preventing and treating ischemic stroke.

**Table 1:** Effect of hormone changes on the prognosis of ischemic stroke.

Hormonal change	data	Prognosis	Reference
Low initial T3	patients	Worse acute ischemic stroke outcomes	(25, 41)
Depressed TSH	patients	Higher risk of post-stroke fatigue	(26)
Low testosterone (in older men)	patients	Increased risk of developing ischemic stroke	(91)
Low testosterone	patients	Associated anger-proneness and emotional incontinence	(94)
Increased testosterone (in the pediatric population)	patients	Elevated risk of stroke	(95)
Lower iPTH (in continuous ambulatory peritoneal dialysis patients)	patients	Increased risk of stroke	(146)

**Table 2:** Effects of hormones on ischemic stroke.

Hormone/Hormone derivative	Tissue/Cell	data	Mechanism	Result	Reference
T3	Brain	animals	Suppresses the expression of aquaporin-4 (AQP4) water channels	Anti-edema and reduction of infarct size	(27)
T3	Hippocampal CA1 region	animals	Increases the neurotrophic factors (BDNF, GDNF)	Significantly improved learning and memory	(28)
T3	Brain	animals	Homeostatic mechanisms regulating the excitability-inhibition ratio in the post-ischemic brain	Enhanced recovery of lost neurological functions	(29)
T3	Astrocytes	animals	Stimulates oxidation of fatty acids and increases the formation of ATP	Increased astrocyte survival	(30)
rT3	Brain	animals	Induces a hypometabolic state of the brain	Reduced markers of neuron injury, infarct size, and neurological deficits	(31)
T1AM	Brain	animals	Induces hypothermia	Less infarct area	(46)
MR-409	Brain	animals	Enhances proliferation of neural stem cells Inhibits apoptosis Stimulates endogenous neurogenesis Improves loss of neuroplasticity Activates AKT/CREB and BDNF/TrkB pathways	Enhanced endogenous neurogenesis and neuroprotection	(56)
GH	Brain	animals	Increases cell proliferation, neurogenesis, synaptic plasticity, and angiogenesis in the peri-infarct region	Decreased infarct size and improved motor function	(63)
GH	Hippocampus	animals	Increases GLUR1 receptor protein	Enhanced hippocampal plasticity and cognitive recovery	(90)
Testosterone	dentate gyrus	animals	suppressed maturation of newborn neurons	Reduced cellular repair in injured	(100)
Estrogen and progesterone	Cerebral cortex	animals	Up-regulates calpain-1 and activates caspase-3	Reduced neurological deficits and infarct volume	(109)
Estrogen and progesterone	Brain	animals	Increases the expression of GLT-1 and EAAT3	Increased behavioral scores and reduced infarct volume reduced	(110)
Estrogen and Progesterone	Brain	animals	Increases Hsp27 phosphorylation	Decreased astrocytosis and increased neuron survival	(111)
17β-estradiol and Progesterone	Brain	animals	Selectively reduces AIM2 and NLRC4 in primary cortical astrocytes and microglial cells	Decreased infarct sizes and neurological impairments	(112)
17β-estradiol and Progesterone	Cortices	animals	Attenuates proinflammatory chemokines CCL2, CCL5, and interleukin 6 Significantly reduces local expression of microglia/macrophage/lymphocyte markers (Iba1, CD68, and CD3)	Reduced cortical infarct area and promoted the recovery of motor sensory function	(113)
17β-estradiol and progesterone	Brain	animals	Inhibits the increase in the miR-375 target genes Bcl-2 and RAD1 Reverses the miR-223 regulated target genes and reduces NR2B and GRIA2	Indirect control of pro-apoptotic and-inflammatory gene translation	(114)
Prolactin	Brain	animals	Reduces the levels of the neurotransmitters, cerebral calcium, and nitrate	Reduced cerebral infarct, brain water content Restored physiological conditions	(128)
Oxytocin	Cerebral cortex and striatum	animals	Decreases Calpain-1 expression Reduces the apoptosis of neurons	Reduced infarct volume	(117)
Oxytocin	Brain	animals	Inhibits apoptotic and NF-κB signaling pathways and increases the expression of VEGF, AQP4, and BDNF proteins	Reduced BBB leakage and infarct size and improved spatial memory function	(118)
Oxytocin	CA1, CA3, and dentate gyrus	animals	Reduces cell death, apoptosis, and inflammatory mediators TNFα and IL-1β	Reduced ischemic damage and improved neurological function and spatial memory	(119)
Dexamethasone	Brain	animals	Inhibits NF-κB p65 expression Suppresses the expression of iNOS, COX-2, TNF-α, and IL-1β	Reduced infarct size and improved neurological deficits	(141)
GR siRNA	Brain	animals	Enhances the expression levels of proinflammatory cytokines (IL-6, IL-1β, and TNF-α) Suppresses BDNF/TrkB signaling	Increased infarction size and neurobehavioral deficits exacerbated	(142)
PTH	Brain	animals	Promotes the expression of nutrient regeneration factors (VEGF, SDF-1, BDNF) Induces the generation of blood vessels Increases the migration and generation of nerve cells	Promoted recovery of sensory and motor functions	(147)
PTHrP	Cortex	animals	Dilates the arterioles and increases blood flow to the ischemic area	Reduced cortical infarct size	(149)
Levodopa	Brain	animals	Expresses D1R, D2R, dopamine, and cAMP-regulated neuronal phosphoproteins in areas around infarction in astrocytes	Enhanced recovery of sensory and motor functions	(154)
Levodopa	Brain	animals	Down-regulates the Nogo-A-positive oligodendrocyte number, Nogo-A, and Nogo-A receptor levels Increases the number of oligodendrocyte transcription factor 2 positive cells	Increased plasticity	(160)
Levodopa/ Carbidopa	Striatum	animals	Decreases proinflammatory cytokines levels and oxidative stress	Ameliorated neurological deficits and reduced infarct size	(161)

**Table 3:** Mechanism of D1R and D2R/D3R agonists and antagonists on ischemic stroke.

	Drug	Agonist or antagonist	Tissue/Cell	Mechanism	data	Result	Reference
D1R	D1R agonist	Agonist	Striatal	Activates PKA and adenosine A1 receptors	animals	Reduced excitatory synaptic transmission	(175)
	(R)-(+)-SKF-38393 hydrochloride	Agonist	Reactive astrocytes	Upregulates GDNF levels	animals	Enhanced recovery of lost brain function	(176)
	SCH23390	Antagonist	Brain	Decreases ischemia-induced upregulation of endogenous tPA	animals	Reduced BBB injury	(177)
D2/D3R	Bromocriptine	Agonist	Hippocampal CA1 neuron	Decreases copper/zinc superoxide dismutase and manganese superoxide dismutase	animals	Reduced neuronal damage	(180)
	Sino	Agonist	Astrocytes	Upregulates DR2/CRYAB expression	animals	Suppressed neuroinflammation	(181)
	Pramipexole	Agonist	Brain	Reduces levels of mitochondrial ROS and Ca^2+^ Elevates the mitochondrial membrane potential and mitochondrial oxidative phosphorylation Inhibits the transfer of cytochrome *c* from mitochondria to cytosol Inhibits the mitochondrial permeability transition pore	animals	Neurological recovery	(182)
	Sumanirole	Agonist	Brain	Reduces mitochondrial reactive oxygen species production Increases mitochondrial membrane potential Protects mitochondrial complex activity	animals	Reduced infarct size Enhanced neuronal survival	(183)
	Haloperidol	Agonist	Brain	Antagonizes the effects of D2/D3 receptor activation	animals	Abolished protective effects of IPC	(184)

Hormones, hormone derivatives, hormone receptors, and hormone combinations should be the focus of future studies. Hormone research has significantly advanced in preclinical studies of ischemic stroke, and most results are beneficial. However, the use of hormones in the clinical management of ischemic stroke is scarce, and the available results present a contradictory picture because of the complexity of the brain injury process in ischemic stroke. Recently, pyroptosis have attracted more and more attention in the study of cerebral ischemia (185). However, there are few studies on the relationship between hormones and pyroptosis in ischemic stroke. Studies have shown that hormone enhances the therapeutic effect of plasma exosomes against cerebral Ischemia-Induced pyroptosis through the Toll-like receptors/nuclear factor kappa-B (TLR/NF-κB) Pathway (186). Other modes of cell death besides apoptosis, such as ferroptosis and necroptosis, may be a good area for further research. In conclusion, we need to explore the mechanisms of brain damage in ischemic stroke and provide methods for treating and preventing ischemic stroke.

## Author contributions

XK and SZ contributed design of the study and manuscript editing. SH wrote the first draft of the manuscript. LL and XT helped prepare the manuscript and collected the data. All authors contributed to the article and approved the submitted version.

## Funding

The National Science Foundation of China supported this study under Grant No.81971008.

## Conflict of interest

The authors declare that the research was conducted in the absence of any commercial or financial relationships that could be construed as a potential conflict of interest.

## Publisher’s note

All claims expressed in this article are solely those of the authors and do not necessarily represent those of their affiliated organizations, or those of the publisher, the editors and the reviewers. Any product that may be evaluated in this article, or claim that may be made by its manufacturer, is not guaranteed or endorsed by the publisher.
